# Comparing risk prediction models aimed at predicting hospitalizations for adverse drug events in community dwelling older adults: a protocol paper

**DOI:** 10.21203/rs.3.rs-2429369/v1

**Published:** 2023-01-18

**Authors:** Michelle S. Keller, Nabeel Qureshi, Elaine Albertson, Joshua Pevnick, Nicole Brandt, Alex Bui, Catherine A. Sarkisian

**Affiliations:** Cedars-Sinai Medical Center; Cedars-Sinai Health System; Cedars-Sinai Health System; Cedars-Sinai Health System; University of Maryland School of Pharmacy; David Geffen School of Medicine: University of California Los Angeles David Geffen School of Medicine; David Geffen School of Medicine: University of California Los Angeles David Geffen School of Medicine

**Keywords:** Drug adverse events, polypharmacy, older adults, medications, primary care

## Abstract

**Background:**

The objective of this paper is to describe the creation, validation, and comparison of two risk prediction modeling approaches for community-dwelling older adults to identify individuals at highest risk for adverse drug event-related hospitalizations. One approach will use traditional statistical methods, the second will use a machine learning approach.

**Methods:**

We will construct medication, clinical, health care utilization, and other variables known to be associated with adverse drug event-related hospitalizations. To create the cohort, we will include older adults (≥ 65 years of age) empaneled to a primary care physician within the Cedars-Sinai Health System primary care clinics with polypharmacy (≥ 5 medications) or at least 1 medication commonly implicated in ADEs (certain oral hypoglycemics, anti-coagulants, anti-platelets, and insulins). We will use a Fine-Gray Cox proportional hazards model for one risk modeling approach and DataRobot, a data science and analytics platform, to run and compare several widely used supervised machine learning algorithms, including Random Forest, Support Vector Machine, Extreme Gradient Boosting (XGBoost), Decision Tree, Naïve Bayes, and K-Nearest Neighbors. We will use a variety of metrics to compare model performance and to assess the risk of algorithmic bias.

**Discussion:**

In conclusion, we hope to develop a pragmatic model that can be implemented in the primary care setting to risk stratify older adults to further optimize medication management.

## Introduction

The use of unnecessary and/or potentially harmful medications is a serious problem in older adults. Four out of ten adults 65 years and older take medications which do not provide much benefit or have significant side effects.(1) *Polypharmacy* – the daily use of multiple prescription and non-prescription medications to manage several chronic diseases – is a related concern. Forty percent of older American adults take 5 or more medications daily,(2) increasing their risk for adverse drug reactions such as falls, bleeding, hospitalizations, and death.(3) Concerns about polypharmacy and its adverse outcomes has led to the recent development of quality measures such as the 2022 Healthcare Effectiveness Data and Information Set (HEDIS) benzodiazepine deprescribing measure.(4) HEDIS measures are used by more than 90% of U.S. health plans.(5) Inclusion of deprescribing measures in quality measure sets reflects an increasing recognition by health system and insurance leaders of the importance of carefully monitoring potentially inappropriate medication use and implementing interventions to manage this clinical issue.

*Deprescribing*, or stopping or tapering potentially inappropriate medications, has been hypothesized to improve quality of life, to reduce falls and fall-related hospitalizations, and reduce healthcare utilization in older adults.(6) However, the evidence on deprescribing interventions for community-based older adults has been mixed.(7) A systematic review and meta-analysis found comprehensive medication reviews may result in small reductions in mortality (OR: 0.74, 95% CI: 0.58–0.95), but found no effects from other types of deprescribing interventions,(8) and found no clear delineating of which patients would benefit the most. Additionally, the review found no significant effects of deprescribing interventions on hospitalizations or emergency department (ED) visits – outcomes important to health systems, older adults, and payors.(8)

As deprescribing medications can be time consuming and challenging, researchers have noted an important gap in identifying *which* patients would most benefit from deprescribing.(9, 10) With limited resources available to address polypharmacy among community-dwelling adults in the primary care setting, physicians, pharmacists, and health system managers have to prioritize which patients should receive various intensities of deprescribing interventions.(10) These could include, at the least intensive level, a comprehensive annual medication review by a pharmacist or clinical decision support advising discontinuation, and at the most intensive level, enrollment in a deprescribing clinic with multiple follow-up visits. While prior studies have used markers for identifying older adults at high risk of polypharmacy-related adverse events such as the number of medications taken every day (e.g., ≥ 5 or ≥ 10 medications), adding additional clinical data such as renal, liver, and cognitive function; comorbidities, age, and other risk factors could make for more precise patient selection for deprescribing interventions. Moreover, combining clinical data with social determinants of health, such as dual eligibility for Medicare-Medicaid, Limited English Proficiency (LEP), living status (e.g., living alone), could help to identify older adults with polypharmacy who have additional need for medication-related support.

Risk prediction models in medicine have been used for allocations to a variety of interventions, including models aimed at identifying individuals at higher risk of breast cancer for referral to genetic counseling,(11) screening patients for lung cancer,(12, 13) assessing risk of stroke for provision of lifestyle interventions,(14) targeting patients at high risk of suicide, (15) and identifying hospitalized patients at high risk of delirium for intervention.(16) Several risk prediction models have been developed for predicting adverse drug reactions and hospitalizations for adverse drug events.(17–21) A 2014 systematic review identified four models aimed at predicting adverse drug events (ADEs) or adverse drug reactions (ADRs), finding several limitations of the existing models, including predictor variables not easily quantifiable or defined, low statistical power available to detect events, and no evidence of impact or implementation of these models.(20) Moreover, while identifying ADEs/ADRs is important, these outcomes may be less important to patients, health systems, and payors compared to hospitalizations or ED visits. A review of the literature identified two models for predicting ADR-related hospitalizations among community-dwelling older adults.(17, 18) However, shortcomings of these models include the inclusion of predictors in the model not readily available in the electronic health record (such as functional status and health-related quality of life scales obtained from a clinical trial)(17) and identification of risk factors only among hospitalized community-dwelling patients and not a broader population.(18) Thus, there is a need for a risk prediction model that could be used in the primary care setting for allocation to different intensities of deprescribing interventions which uses outcomes important to patients and payors and employs easily available and updated data. Importantly, measures used in the predictor model should be pragmatic and easily found in administrative data (such as electronic health record data) for reproducibility in other health systems and settings. Moreover, ideally, to facilitate implementation, a practical risk prediction model should incorporate existing pharmacy and healthcare utilization measures that health systems may already be tracking or may use in the future, increasing the likelihood of implementation.

Most risk prediction models have employed traditional statistical methods such as logistic regression to predict the risk of a binary outcome. This approach uses a set of clinical, demographic, and other variables selected based on assumptions and prior literature to predict the outcome. As Goldstein *et al*. have noted, limitations of these approaches include the potential for a lack of linearity between the risk factors and the outcome, which may not be correctly modeled in a simple risk prediction model; heterogeneity of effects due to interactions; and a limit on the number of prediction variables.(22) However, newer approaches to risk prediction have used machine learning methods.(22) Advantages include the ability to include more predictors, to more easily incorporate non-linearity, and to mitigate missing data without imputation.(22) Limitations include the potential for incorporating and amplifying existing racial biases (see below), the lack of an easily clinically interpretable relationship between the predictors and outcome, issues incorporating temporal data, and challenges creating risk scores that can be meaningfully used in clinical applications when using some machine learning approaches.(22) Machine learning models may also not perform any better than models using traditional statistical methods, highlighting the importance of comparing both approaches.

An important consideration in the use of machine learning models is that they may amplify existing racial, ethnic, gender, and other biases.(23) There are known biases in healthcare access and delivery such as differential treatment of pain,(24) timeliness to radiography and surgery,(25, 26) use of physical restraints in the emergency setting,(27)among many other examples. Race corrections in clinical algorithms, such as the estimate glomular filtration rate (eGFR), have recently been under scrutiny for exacerbating racial and ethnic disparities.(28) Incorporating clinical and healthcare utilization variables into any algorithm – machine learning or otherwise – carries a risk of integrating these existing biases into the algorithm. Thus, integrating a variety of “fairness metrics” can help identify whether different groups are treated equally in the algorithm.(29)

The objective of this paper is to describe the creation, validation, and comparison of two risk prediction modeling approaches for community-dwelling older adults to identify individuals at highest risk for adverse drug event-related hospitalizations. One approach will use traditional statistical methods, the second will use a machine learning approach. We used the Transparent Reporting of a multivariable prediction model for Individual Prognosis or Diagnosis (TRIPOD) checklist and the PROBAST (Prediction model Risk Of Bias Assessment Tool), a tool for assessing the risk of bias to guide our methods.(30, 31) If successful, the risk prediction model will be used to guide a deprescribing strategy at the health system level.

## Methods

### Data sources

For model development, we will include older adults (≥ 65 years of age) empaneled to a primary care physician within the Cedars-Sinai Health System primary care clinics (~ 20,000 patients) with polypharmacy (≥ 5 medications) or at least 1 medication commonly implicated in ADEs (some oral hypoglycemics [sulfonylureas], anti-coagulants [warfarin and direct-acting oral anticoagulants], some anti-platelets [platelet P2Y12 receptor agonists] and insulins).(32–34) We selected to focus on empaneled patients given that the majority of their healthcare utilization would be captured within the health system’s electronic health records. While this may potentially limit generalizability to patients in non-managed care plans, for this study, we opted to prioritize internal validity. We will use data from the electronic health record warehouse, which includes demographics, diagnoses, lab values, and medications. In future studies we will test the external validity of the CSMC-derived model with datasets from other health systems (including those with more non-managed care patients).

### Outcome

The primary outcome is a composite measure consisting of any adverse-drug related ED visit or hospital admission in the next year. We used a list of the most common ICD-10 codes used to identify adverse drug events in administrative data from a systematic review by Hohl et al.(35)

#### Cohort creation:

To create the cohort, we will include older adults (≥ 65 years of age) empaneled to a primary care physician within the Cedars-Sinai Health System primary care clinics with polypharmacy (≥ 5 medications) or at least 1 medication commonly implicated in ADEs (some oral hypoglycemics [sulfonylureas], anti-coagulants [warfarin and direct-acting oral anticoagulants], some anti-platelets [platelet P2Y12 receptor agonists] and insulins).(32–34) We will create several cohort years from 2016–2021. For each year, we will select the closest encounter date on or before January 1 for all patients. For example, for the year 2016, we will select the closest encounter date on or before January 1, 2016. We will obtain diagnoses and clinical values one year prior to this index encounter date from any encounter. To obtain the outcome, we will look one year forward from this index date. We will obtain electronic health records including all encounters at the health system, including outpatient and inpatient encounters.

### Logistic regression risk prediction model

#### Predictive variables for ADR-related hospitalization.

We will perform preliminary screening for collinearity using pairwise correlations between all predictors and will drop a predictor if the correlation is greater than 0.75.(36) Following best practices for risk prediction models, we will use multiple imputation for missing data.(36)

### Medications and medication-related measures

- We will include medications identified as important based on the Age-Friendly Health Systems (AFHS) Framework and Pharmacy Quality Alliance.(37–39) These medications include those aimed at preventing, treating, and managing dementia, depression, and delirium across settings of care. Medications that should be reviewed as part of the AFHS framework include benzodiazepines, opioids, central nervous system (CNS) depressants, highly anticholinergic medications (e.g., diphenhydramine), prescription and over-the-counter sedatives, muscle relaxants, antipsychotics, and tricyclic antidepressants.(38) We will operationalize these medications in the following ways:
  - For benzodiazepines, we will convert the monthly dose to a diazepam daily equivalent and calculated the mean monthly dose. We will use diazepam dose equivalent conversion factors from Borrelli et al.(40)
  - For opioids, we will use measures from the Pharmacy Quality Alliance,(41) and create dummy variables if patients meet the following:
    - Use of opioids at high dosage in persons without cancer, defined as ≥ 120 morphine milligram equivalents for ≥ 90 consecutive days;
    - Multiple prescribers, defined as receiving opioid prescriptions from ≥ 4 providers and ≥ 4 pharmacies within 180 days (excluding patients with cancer, sickle cell disease, or receiving palliative care);
    - Concurrent use of opioids and benzodiazepines, defined as having ≥ 30 cumulative days of overlapping opioids and benzodiazepines among individuals having ≥ 2 opioid prescriptions and ≥ 2 benzodiazepine prescriptions.
  - For highly anticholinergic medications (e.g., antihistamines, antiparkinsonian agents), we will create a dummy variable defined as concurrent use for ≥ 30 days or ≥ 2 unique anticholinergic medications.(42)
  - For CNS depressants, we will create a dummy variable defined as concurrent use for ≥ 30 days or ≥ 3 unique CNS medications (including: antipsychotics, benzodiazepines and non-benzodiazepine sedative-hypnotics, opioids, selective serotonin reuptake inhibitors, serotonin-norepinephrine reuptake inhibitors, tricyclic antidepressants, and antiepileptics).(43)
  - For prescription and over-the-counter sedatives and sleep medications (recorded in the electronic health record), we will create a dummy variable if a patient had ≥ 30 days supply.
  - For muscle relaxants, we will create a dummy variable if a patient had any prescriptions within the last year.(38)
  - Tricyclic antidepressants, we will create a dummy variable if a patient had ≥ 30 days supply within the last year.(38)
  - For antipsychotics, we will create a dummy variable defined as at least one prescription and ≥ 30 days supply for any antipsychotic medication and no diagnosis of schizophrenia, bipolar disorder, Huntington’s disease, or Tourette’s syndrome.(39)
- A variable for the maximum count of all medications (over-the-counter and prescription) at any one encounter.

### Demographics

We will include, age, sex (as a biological variable, as there may be sex-based differences in drug metabolism), race, ethnicity, interpreter needed (yes/no), primary language (English vs. not English), marital status (as a proxy for social support), and insurance status (Medicare Advantage, Medicare, or Medicare-Medicaid).

### Diagnoses

In addition to including the Charlson Comborbidity Index (CCI) as a cumulative measure of multimorbidity burden,(44) we will include dummy variables for the following clinical diagnoses previously used in ADE-prediction models: depression, osteoarthritis, heart disease, hypertension, hyperlipidemia, cancer, chronic obstructive pulmonary disease, incontinence, congestive heart failure, substance use disorder.(18) We will use documented diagnoses in the electronic health record from the 1 year before the baseline date.

As dementia is often underdiagnosed and underreported, we will de ne dementia as either a diagnosis of dementia using the Bynum-standard algorithm and/or ≥ 2 prescriptions and ≥ 60 days’ supply of a cholinesterase inhibitor or a N-methyl-D-aspartate (NMDA) receptor antagonist.(45) The Bynum-standard algorithm uses the following ICD-10 codes to identify dementia: F01.50-F01.51 (vascular dementia), F02.80-F02.81 (dementia), F03.90-F03.91 (unspecified dementia), F04 (amnestic disorder), G30.0/30.1/30.8/G30.9 (Alzheimer’s disease), G31.01 (Pick’s disease), G31.09 (frontotemporal dementia), G31.83 (dementia with Lewy bodies), G31.1 (senile degeneration), G31.2 (degeneration of nervous system), R41.81 (age-related cognitive decline).(46)

### Clinical values

We will include clinical measures known to be associated with drug metabolism and potential adverse effects, including:

- Depression, as measured by the PHQ-2 and PHQ-9
- Glomular filtration rate (eGFR),(47) calculated using serum creatinine, serum cystatin C, age, and sex
- Liver function, as calculated via the MELD 3.0 (Model for End-Stage Liver Disease) Score.(48) The MELD is used primarily to stratify individuals based on severity for transplant purposes, though it has been used for other purposes. The score ranges from 6 to 40, with higher numbers associated with higher severity. The score is derived from the following variables: Whether a patient is on dialysis, Creatinine, Bilirubin, Internal Normalized Ratio (INR), Serum Sodium(48)
- Hemoglobin concentration(49)
- Mean and minimum systolic and diastolic blood pressure(50)
- Mean and minimum pulse(50)
- Mean and maximum body temperature(51)

### Healthcare utilization

We will also control for healthcare utilization throughout the previous year to account for informed presence bias. We will control for the number of outpatient and inpatient encounters in the last year.(52)

### Social determinants of health screening

In January 1, 2021, Cedars-Sinai Medical Center began administering a social determinants of health screener to patients in the acute care setting and in some non-acute settings. We will use variables recorded in the electronic health record that measure social determinants of health, understanding that there may not be data available for all individuals in our sample, including:

- Food insecurity: (“Within the past 12 months, you worried that your food would run out before you got the money to buy more”; response choices: never true, sometimes true, often true, no response)
- Transportation needs: (“In the past 12 months, has lack of transportation kept you from medication appointments, from getting medications, getting things needed for daily living?”; response choices: yes, no, no response)
- Financial resource strain (“How hard is it for you to pay for the very basics like food, housing, medical care, and heating?”; response choices: very hard, hard, somewhat hard, not very hard, not hard at all, no response)
- Access to care (“In the last 12 months, have you needed to see a doctor but could not because of cost or insurance issues?”; response choices: hardly ever, some of the time, often, no response)
- Health literacy (“Do you ever need help reading or understanding information about your medical condition?”; response choices: yes, no, no response
- House stability (“What is your housing situation today?”; response choices: I have housing, I do not have housing, no response. Also, “Are you worried about losing your housing?”; response choices: yes, no, no response)
- Independent living (“Because of a physical, mental, or emotional condition, do you have difficulty doing activities or errands alone such as visiting a doctor’s office or shopping?”; response choices: hardly ever, some of the time, often, chose not to respond)
- Social connections (“How often do you lack companionship?”, “How often do you feel isolated from others?”, “How often do you feel left out?”; response choices: hardly ever, some of the time, often, chose not to respond)

As these variables are not available in every health system and the responses may not be standardized across different health systems, we will estimate models with and without this set of variables to ensure generalizability and potential dissemination of the model. We will also determine the level of missingness of these variables and consider whether multiple imputation is needed.

#### Statistical analysis.

We will first use chi-square or t-tests to compare characteristics of individuals who did and did not experience an ADE-related hospitalization or ED visit within the year.

We will use a Fine-Gray Cox proportional hazards model(53) which accounts for competing risks to predict the time to an ADE-related hospitalization or ED visit. Competing risks will include mortality or a non-ADE-related hospitalization/ED visit. We will use the Stata function *stcrreg* function, which allows for multiple-record data (in this case, multiple observations per patient across the different cohort years). We will use robust standard errors.

For validation, we will use a bootstrap validation approach, where we will construct 200 bootstrap datasets by randomly sampling patients with replacement and will fit the model to each bootstrap dataset.(54) Estimated coefficients will be used to obtain predictions for patients in the original prediction model, which will be used to calculate the calibration slope for the fitted model.(54)

### Machine learning risk prediction algorithms

We will use DataRobot, a data science and analytics platform, to run and compare several widely used supervised machine learning algorithms, including Random Forest, Support Vector Machine, Extreme Gradient Boosting (XGBoost), Decision Tree, Naïve Bayes, and K-Nearest Neighbors.(55) DataRobot is an automated machine learning platform that can rapidly compare model types and tune hyperparameters to optimize prediction accuracy. Automated machine learning with DataRobot, and other platforms, has emerged as part of “state-of-the-art” healthcare analytics (Waring 2020). Prior studies have used DataRobot for healthcare risk prediction.(56–58) The cohort selection will use the same process as above. We will use the same constructed measures as above, with the exception of the following:

- We will construct several summary-level values (means, minimum, maximum, standard deviation) for laboratory values.
- We will include all diagnoses in the year prior to the index date.

As described above, inadvertent algorithmic bias in ML is a recognized problem. We will assess data and algorithms through population stratification and quantitation of bias following three paths:(59)

- **Dataset representation.** It is important to determine to what extent the data used to train an algorithm comes from a population that is representative for its intended use (in this case, the overall empaneled population in the health system). We will assess statistical distributions, and also determine the levels of associated missingness. If a particular subgroup is underpowered and/or has a significant amount of missing data in each model feature, we will look to apply inverse-odds weighting/transportability as cited above to accordingly compensate.
- **Stratification and subgroup analyses.** We will calculate group benefit equality (GBE), an algorithmic fairness metric that quantifies the rate at which a particular event is predicted to occur within a subgroup compared to the rate at which it actually occurs; and apply the fairness principles which examine the equality of opportunities for various subgroups.(59) The use of the GBE can help identify whether a particular subgroup (e.g., a racial or ethnic subgroup) is being flagged for intervention at a lower rate than the prevalence of the event in the subgroup – in this case, because the predicted probability of ADE-related hospitalization is potentially incorrectly or biasedly under-predicted.
- **Ablation studies.** We will selectively remove and replace variables to ascertain any impact on guiding a predictive model’s results, evaluating the relative impact.

From these results and application of the Prediction model Risk Of Bias Assessment Tool (PROBAST),(60) we will determine if our models are prone to potential bias and adapt the models accordingly to address these issues.

### Model comparison

We will use a variety of performance metrics to compare the various models. These include:

- the Brier score, or squared-loss error, which takes the difference between the model’s predictions and the actual outcomes;
- the misclassification rate;
- the c-statistic, which measures the model’s ability to correctly classify those who have or do not experience an ADE-related hospitalization or ED visit based on predicted risk and is analogous to the area under the receiver operating characteristic curve, a plot of sensitivity versus (1–specificity) for a binary outcome at various thresholds;(36)
- Area under the precision-recall curve, which summarizes precision and recall;
- calibration plots where the mean predicted probability of the outcome is plotted against the observed proportion of outcomes for groups of the cohort;(36)
- the Hosmer-Lemeshow test;(36)
- the log loss or logarithmic score;
- the Gini coefficient, which measures a model’s discriminatory power.

We will also create a confusion matrix to examine positive and negative predictive values and sensitivity and specificity for each model. Finally, we will conduct error analysis to descriptively examine the errors that each model makes (for example, comparing diagnostic/clinical/demographic characteristics for the patients correctly vs. incorrectly classified in each model).

For the machine learning algorithms, we will also compare variable importance for each final model. We will also examine any potentially bias by examining the proportionate number of people in different racial and ethnic categories identified as higher risk.

## Discussion

Deprescribing interventions may require large amounts of time from physicians and pharmacists. Identifying which patients may benefit the most from deprescribing interventions has the potential to improve efficiencies and workflow.. We have described a rigorous a *priori* methodology to create and validate a disseminatable tool that can use EHR data to identify older adults most likely to benefit from deprescribing interventions.

Limitations of this approach are similar to other models using administrative data such as electronic health records. For example, there may be miscoding, over-coding, or under-coding of diagnoses, which may introduce bias. For example, it is well known that dementia is under-diagnosed due to structural factors (e.g., lack of knowledge, resources, training, and time among primary care providers to diagnose dementia).(61) Moreover, an important consideration is that access to healthcare may be impeded by structural factors such as lack of transportation, insurance, language issues, discrimination and racism, etc. This issue may result in under-diagnosed conditions and lower quality of care experienced among patients from vulnerable and marginalized patients.(62) These populations may also have fewer encounters or fewer laboratory and test values, which could result in an underestimation of risk among these patients. As a result, it will be important to examine the demographic characteristics of individuals classified in the various models to explore bias in these models.

In conclusion, we hope to develop a pragmatic model that can be implemented in the primary care setting to optimize medication management in older adults.

## Data Availability

Not applicable
